# Emerging cardiovascular risk factors in childhood and adolescence: a narrative review

**DOI:** 10.1007/s00431-025-06102-y

**Published:** 2025-04-14

**Authors:** Ana De Blas-Zapata, Jose Manuel Sastre-Albiach, Laura Baixauli-López, Rocío López-Ruiz, Julio Alvarez-Pitti

**Affiliations:** 1 Pediatric Department, Consorcio Hospital General, University of Valencia, 46014 Valencia, Spain; 2 Innovation in Paediatrics and Technologies-iPEDITEC-Research Group, Fundación de Investigación, Consorcio Hospital General, University of Valencia, Valencia, Spain; 3 CIBER Fisiopatología Obesidad y Nutrición (CIBEROBN), Instituto de Salud Carlos III, 28029 Madrid, Spain

**Keywords:** Children, Adolescence, Cardiovascular risk factors, Hypertension, Psychosocial factors

## Abstract

It is widely recognized that four key health behaviors—regular physical activity, maintaining a normal BMI, eating a healthy diet, and avoiding smoking—offer significant protection against cardiovascular disease in children and adolescents. However, changes in the lifestyle of families have contributed to the emergence of new behaviors that may impact the health of children and adolescents. This narrative review aims to identify existing evidence on the effect of these arising habits on the cardiovascular health of children and adolescents, mainly on blood pressure and endothelial function. A thorough search was conducted across various databases, including PubMed/MEDLINE, the Cochrane Library, Science Direct and EBSCO.

*Conclusion: *Some of the behaviors most frequently identified in the pediatrician’s office are childhood stress and behavioral disorders, new forms of nicotine consumption, the impact of the use of screens and digital devices, changes in sleep patterns, and, finally, the generalization of energy drinks and supplements to promote muscle development, mainly in adolescents. The effect on cardiovascular health, mainly on blood pressure, does not seem negligible. Early identification of these unhealthy behaviors might allow the pediatrician to intervene and prevent the progression of cardiovascular disease.

**Table:** 

**What is Known:** • *Traditional cardiovascular risks (poor diet, inactivity, smoking, obesity) contribute to hypertension and endothelial dysfunction in youth.* • *Western family lifestyles have shifted dramatically over two decades, altering pediatric environments.*	
**What is New:** • *Emerging risks include psychosocial stressors, novel nicotine products, screen time-induced HTN, sleep deprivation, and energy drink/supplement use.* • *These factors correlate with blood pressure elevation, endothelial damage, and chronic inflammation, urging pediatricians to address non-traditional factors in holistic care.*

**What is Known:**

• *Traditional cardiovascular risks (poor diet, inactivity, smoking, obesity) contribute to hypertension and endothelial dysfunction in youth.*

• *Western family lifestyles have shifted dramatically over two decades, altering pediatric environments.*

**What is New:**

• *Emerging risks include psychosocial stressors, novel nicotine products, screen time-induced HTN, sleep deprivation, and energy drink/supplement use.*

• *These factors correlate with blood pressure elevation, endothelial damage, and chronic inflammation, urging pediatricians to address non-traditional factors in holistic care.*

## Introduction

Primordial prevention focuses on preventing the development of cardiovascular risk factors from the very beginning, while primary prevention involves treating these risk factors to prevent cardiovascular disease. Most children are born with ideal cardiovascular health. However, modifiable risk factors such as poor diet, physical inactivity, and smoking can progressively impair cardiovascular function. To preserve ideal cardiovascular health, the American Heart Association (AHA) proposed seven cardiovascular health metrics in 2010 for both children and adults [1]. These metrics encompass four health behaviors [non-smoking, regular physical activity, maintaining a normal body mass index (BMI), and eating a healthy diet] and three health factors (normal blood pressure (BP), total cholesterol, and plasma glucose levels). It is necessary to meet all seven metrics to have a healthy cardiovascular system. In 2016, the AHA refined these cardiovascular health metrics for children and adolescents, as detailed in Table 1 [2].

**Table 1:** Poor, intermediate, and ideal definitions: health behavior and health metrics in children and adolescents

Metric	Poor	Intermediate	Ideal
Health behavior			
Smoking status	Tried > 30 d ago	…	Never tried; never smoked a whole cigarette
BMI	> 95th percentile	85th–95th percentile	< 85th percentile
Physical activity level	None	> 0 and < 60 min/d moderate or vigorous activity every day	≥ 60 min/d moderate or vigorous activity every day
Healthy Diet Score*	0–1 components	2–3 components	4–5 components
Health factor			
Total cholesterol	≥ 200 mg/dL	170–199 mg/dL	< 170 mg/dL
Blood pressure	> 95th percentile	90–95th percentile	< 90th percentile
Fasting blood glucose	≥ 126 mg/dL	100–125 mg/dL	< 100 mg/dL

Table modified from [2]

*BMI* indicates body mass index

^*^The Healthy Diet Score is based on adherence to the following recommendations: fruits and vegetables, ≥ 4.5 cups per day; fish, ≥ 2 3.5-oz servings per week; sodium, ≤ 1500 mg/d; sugar-sweetened beverages, ≤ 450 kcal (36 oz) per week; and whole grains, ≥ 3 servings a day scaled to a 2000-kcal/d diet

Cardiovascular and metabolic disease risk factors often emerge early, with childhood obesity accelerating their progression [3]. However, in the last 15 years, the lifestyle of families and, therefore, of children and adolescents in Western societies has undergone major changes [4, 5]. As a result, it may be that new risk behaviors have appeared that were not considered when these guidelines were designed and that not only increase the risk of cardiovascular and metabolic disease by favoring the development of obesity but also by affecting cardiovascular health through other mechanisms.

This narrative review, including English-language meta-analyses, systematic reviews, randomized clinical trials, and observational studies, aims to identify emerging risk behaviors widespread among children and adolescents that could threaten their cardiovascular health, focusing on the impact on blood pressure and endothelial function. Articles were selected by consensus of all authors. The literature search was conducted using a combination of the following keywords: “cardiovascular risk factors,” “hypertension,” “adverse childhood experiences,” “psychosocial stressors,” “mood disorders,” “depression,” “tobacco exposure,” “nicotine consumption,” “combustible tobacco products,” “non-combustible tobacco products,” “electronic tobacco products,” “screentime,” “sedentary behavior,” “sleep disturbances,” “sleep apnea syndrome,” “sleep deprivation,” “energy drinks,” “sugar-sweetened beverages,” and “muscle-building supplements,” limiting the search to studies including children and adolescents under 18 years old. The search was limited to the last 10 years since this is the period during which these new behavioral risk factors were established among children and adolescents.

After careful evaluation of the existing literature, five changes in the lifestyles of children and adolescents have been identified that may increase the risk of developing hypertension (HTN). These new risk behaviors are changes in the psychosocial environment, new forms of nicotine consumption, the impact of digital devices and sleep deprivation, and finally, energy drinks (EDs) and muscle-building supplements consumption.

By highlighting these behaviors, this review aims to underscore the critical role of pediatricians in the primordial prevention of cardiovascular disease, enabling them to take proactive measures in safeguarding the long-term cardiovascular well-being of their patients.

### Psychosocial risk factors

Adverse childhood experiences (ACEs) encompass a range of potentially stressful and traumatic events or circumstances that occur during childhood and adolescence before the age of 18. These experiences may directly impact the child or alter the broader environment in which they develop. ACEs include but are not limited to, emotional, physical, or sexual abuse, severe accidents or injuries, chronic illness, parental death, and dysfunctional family dynamics. These psychosocial stressors exert their effects during critical periods of neurodevelopment, leading to prolonged activation and maladaptive regulation of allostatic systems, which may result in long-term alterations in physiological stress responses and overall health trajectories with a negative impact on psychological and physical health throughout life [6].

There is growing evidence that these determinants of psychosocial stress influence the risk of HTN among children and adolescents. Previous studies demonstrated that children who experienced physical and/or sexual abuse before the age of 18 [7] or who were separated from their parents during World War II exhibited significantly higher systolic blood pressure (SBP) and diastolic blood pressure (DBP) in adulthood compared to their non-separated counterparts [8]. Socioeconomic adversity in childhood has also been suggested as an important determinant of risk for HTN in adulthood [9]. Notably, children and adolescents exposed to multiple ACEs before age 18 show increases in SBP during young adulthood, independently of BMI, race, risk behaviors, or sex [10].

The precise mechanisms underlying the increased risk of HTN in children and adolescents exposed to ACEs remain not completely understood. However, chronic stress is widely considered a key contributor, with prolonged activation of stress pathways leading to vascular damage and dysfunction associated with ACEs [11]. The elevated concentration of stress hormones and circulating catecholamines has been linked to the development of an inflammatory phenotype [12], hemodynamic changes suggestive of vascular dysfunction [11], increased levels of endothelin-1 [10], and decreased sirtuin-1 levels [13, 14].

Recent research has increasingly focused on the potential epigenetic impacts of ACEs, particularly DNA methylation of key regulatory genes. It may also be related to the shortening of telomeres, accelerating the cellular aging process, and ACEs at younger ages having greater negative effects [13, 14]. These stress-induced epigenetic modifications may have intergenerational effects, although this phenomenon has yet to be conclusively demonstrated in humans [15].

In 2015, the AHA recognized mood disorders, including major depression and bipolar disorder, as moderate risk factors for cardiovascular disease [16]. Since then, numerous cross-sectional and longitudinal studies, despite considerable variability, have supported this association. These studies provide evidence linking mood disorders with elevated DBP and overall HTN in children and adolescents, though no such association has been consistently observed with anxiety disorders [17]. These increases in HTN have not been attributed to a direct effect of antidepressant medications, particularly selective serotonin reuptake inhibitors, which are the most commonly prescribed in the pediatric population, except for venlafaxine [18].

### The changing landscape of nicotine use in adolescents

**Fig. 1:**
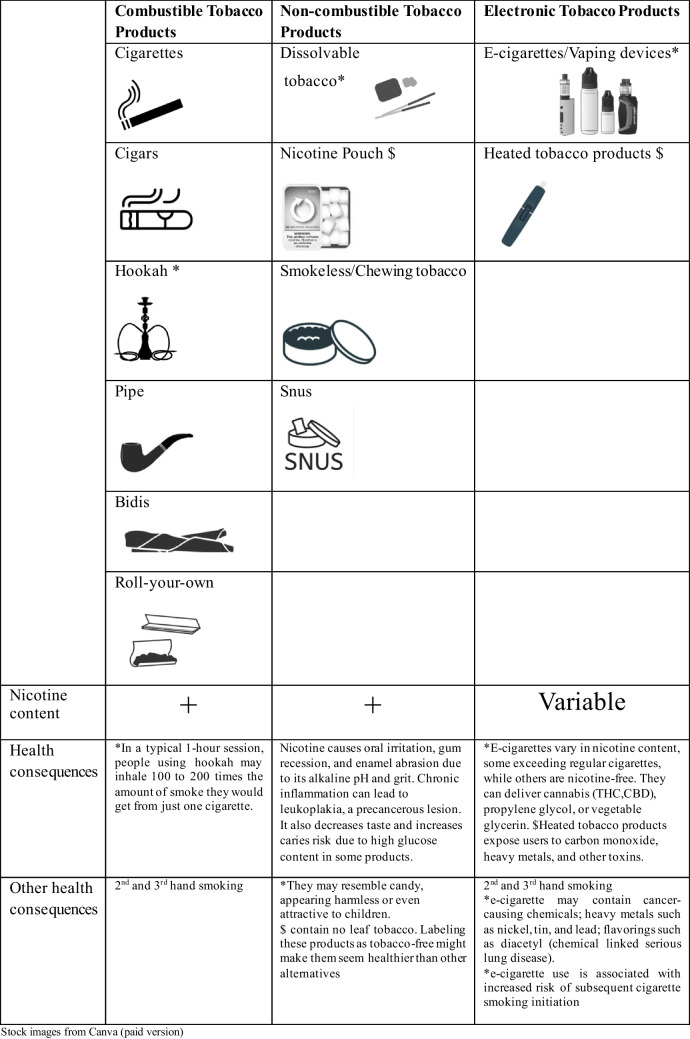
Tobacco consumption methods. Stock images from Canva (paid version)

Over the past 20 years, the number of different tobacco consumption methods has multiplied. These include forms of consumption with or without tobacco combustion and electronic products, as summarized in Fig. 1.

While the consumption of traditional forms of combusted tobacco has declined, alternative forms of consumption have grown in popularity. The data collected in the USA in 2022 revealed that 11.3% of all students were current users of any tobacco product, including 16.5% of high school students and 4.5% of middle school students. E-cigarettes were the most prevalent tobacco product among high school students (14.1%), followed by cigars (2.8%), cigarettes (2.0%), smokeless tobacco (1.6%), hookahs (1.5%), nicotine pouches (1.4%), heated tobacco products (1.1%), and pipe tobacco (0.7%). Among middle school students, e-cigarettes were the most prevalent form of tobacco consumption (3.3%), followed by cigarettes (1.0%), smokeless tobacco (0.7%), heated tobacco products (0.7%), cigars (0.6%), hookahs (0.5%), nicotine pouches (0.5%), and pipe tobacco (0.3%) [19].

According to data from the 2024 National Youth Tobacco Survey (NYTS), e-cigarette consumption has decreased to 7.8% of high school students [19]. Nevertheless, no changes have been observed in middle school students nor in the use of noncombustible tobacco products, as these newer products are often perceived as safer, but they still contain nicotine.

In Europe, the figures are worse. According to the 2021/2022 Health Behavior in School-Aged Children study, approximately one-third (32%) of 15-year-olds in Europe had used e-cigarettes at some point in their lives, and 20% had used them within the previous month. By contrast, 25% of 15-year-olds had smoked a conventional cigarette in their lifetime, and 15% had smoked one in the preceding 30 days.

Nicotine is a highly addictive substance that affects brain development and increases the risk of long-term dependence. The earlier in childhood an individual uses any nicotine-containing product, the stronger the likelihood of developing tobacco use disorder and the more difficult it is to quit [20]. Adolescents’ brain cell activity in the parts of the brain responsible for attention, learning, memory and executive function can be modified by nicotine [21], favoring the development of attention-deficit/hyperactivity disorder, increased risk of mood disorders (anxiety and depression) [20] and long-term effects on the ability to make decisions [22].

Focusing on cardiovascular health effects, nicotine acutely raises blood pressure by activating the adrenergic pathway and triggering epinephrine and norepinephrine release [23]. The link between nicotine exposure and chronic HTN in children and adolescents is less definitive. In the meta-analysis conducted by Jamaati et al., neither active nor passive cigarette smoking was associated with the development of HTN [24]. However, in a recent cross-sectional study including 8520 well-phenotyped children, an association was found between tobacco exposure and elevation of BP, which persisted after adjustment for potential confounders [25]. There is evidence suggesting a potential pathway linking nicotine exposure and the development of HTN through endothelial damage and vasculopathy, and the detrimental effects of tobacco exposure on endothelial function are evident even in young children without other risk factors [26, 27].

Finally, it is worth noting the existence of the well-known effects, mainly at the respiratory level, of the nicotine consumption patterns involving combustion [28].

Considering these premises and knowing the quantity and mechanism of nicotine delivery of each of these new modalities, the cardiovascular effects of each can be estimated, as summarized in Fig. 1.

Faced with this problem, interventions for vaping prevention have been developed in different countries, but with little hopeful effects because school-based interventions showed efficacy in reducing past 30-day tobacco use but not e-cigarette use [29].

### The impact of screen exposure

Currently, children and adolescents are digital natives, and the use of digital devices has increased following the SARS-CoV-2 pandemic, a trend that has persisted to the present day [30]. Major scientific organizations recommend minimizing screen use as much as possible before the age of 2, with a maximum of 1 h per day of supervised content recommended for children aged 2 to 5 years. For children over the age of 5, screen time should be restricted to less than 2 h per day [31–34].

HTN in childhood and adolescence is a multifactorial condition influenced by genetic, physiological, and environmental factors. Among the environmental contributors, prolonged screen time has been implicated as a risk factor for HTN through multiple mechanisms. Evidence from various studies indicates that excessive screen time adversely impacts both the quality and duration of sleep, thereby heightening the risk of HTN, particularly in younger children [35, 36].

Another relevant factor is the sedentary lifestyle associated with prolonged screen use, which negatively impacts cardiometabolic fitness. This leads to reduced arterial elasticity and increased intima-media thickness, both of which have been linked to higher BP levels [37, 38].

Stress associated with using electronic devices is another factor contributing to the development of HTN. The content consumed and the frequent use of these devices can increase sympathetic nervous system tone, leading to a higher risk of arteriolar damage and, ultimately, elevation of BP. This sympathetic activation appears to be more pronounced in males, who also exhibit higher baseline systolic and diastolic BP compared to females, due to their greater stroke volume and total peripheral resistance [39, 40].

A meta-analysis conducted in 2023 revealed that prolonged screen use, defined as > 2 h per day, increased the risk of HTN by 7% and raised SBP by 1.9 mmHg. Additionally, a non-linear correlation was identified between HTN risk and screen time, with the highest risk observed with 100–150 min of daily use of electronic devices [41].

These findings highlight the importance of implementing preventive measures that promote regular physical activity and reduce screen time to prevent the development of HTN and improve cardiovascular health from an early age, as well as to prevent related complications. Parental control and family education could be an effective measure in this regard.

### Sleep disturbances

Sleep disturbances affect an estimated 25–50% of children worldwide, with their prevalence rising steadily [42, 43]. Sufficient sleep is crucial for children’s well-being since various aspects of sleep, such as duration, timing, and quality, are increasingly associated with a range of health outcomes [44]. Over the past century, sleep duration in children and adolescents has gradually declined, contributing to health risks, including cardiovascular disease [45].

Although the physiological role of sleep is not fully understood, recent research has highlighted its association with cardiovascular risk [46]. Sleep disturbances, such as obstructive sleep apnea [47] and chronic sleep deprivation, are associated with increased risk of atherosclerosis and other cardiovascular conditions [48, 49]. These sleep disorders may act as causal factors or important modifiers in the association between cardiovascular risk biomarkers and clinical outcomes.

While inadequate sleep is a known risk factor for childhood obesity, its impact on elevated BP in youth is less clear. A cross-sectional study made in Spanish population, reported a significant inverse correlation between sleep duration and BP, such as in children aged 7–16, where shorter sleep was linked to increased pulse pressure and mean arterial pressure [50]. Numerous studies in pediatric populations summarized in a systematic review have found associations between reduced sleep duration and HTN [51].

Mixed results were observed in certain longitudinal studies, where initial associations between sleep duration and systolic BP at 2 months of age were found. However, this association disappeared by 6 years of age [52]. In a recent systematic review, strong evidence was found linking short sleep duration to increased adiposity and elevated BP [51]. One meta-analysis showed that a 1-h reduction in sleep duration was associated with a 0.33 mmHg increase in SBP and a 0.21 mmHg rise in DBP [53]. Another study found a 51% higher risk of HTN in adolescents with short sleep [54]. However, a previous review has pointed out that these associations may differ based on age, sex, and race, suggesting the need for further research [55].

The mechanisms underlying these associations involve endothelial dysfunction and inflammation. Sleep disruption activates the sympathetic nervous system, raising heart rate, and peripheral vascular resistance, which elevates BP. It also induces low-grade inflammation and alters immune responses, which damage the endothelium [56]. Endothelial dysfunction, characterized by reduced nitric oxide and elevated endothelin-1 levels, impairs vascular relaxation and promotes vasoconstriction. Furthermore, reduced hypocretin levels from sleep loss trigger inflammatory cell production, contributing to vascular inflammation and arterial stiffness [57]. These findings have been supported by research in animal models and confirmed in studies involving humans, linking sleep fragmentation to increased inflammatory white blood cells and atherosclerosis [58].

### Energy drinks and muscle-building supplements

EDs are sugar-sweetened beverages that typically contain a combination of carbohydrates, caffeine, guarana, and taurine. Their consumption is prevalent among adolescents and has been associated with various cardiovascular disturbances, including elevated SBP, cardiomyopathy, and heart palpitations [59].

Mandilaras et al. investigated the acute cardiovascular effects of EDs in healthy children and adolescents, revealing that acute ED consumption was associated with a significant increase in supraventricular extrasystoles and a marked decrease in heart rate likely driven by an acute peak of both systolic and DBP. Although no significant alterations in QTc interval were observed compared to placebo, there was a notable reduction in the QT–RR relationship, suggesting a reflex autonomic response. The current findings raise concerns that children with pre-existing cardiac rhythm disorders may be at risk of malignant arrhythmias following ED consumption [60].

Oberhoffer et al. investigated the effects of a single dose of body weight-adjusted ED on 24-h ABPM in healthy children and adolescents. Their findings indicated that a single dose of ED was associated with a significantly higher median 24-h SBP (+ 5.26 mmHg) and DBP (+ 3.45 mmHg) compared to a placebo drink, suggesting a notable impact on cardiovascular parameters in healthy children and adolescents [59].

Chronic consumption of ED could result in arterial HTN and thus increased left ventricular afterload, ultimately leading to left ventricular dysfunction and hypertrophy [61]. Additionally, acute consumption of ED has been linked to a significant increase in arterial stiffness of the common carotid arteries in healthy children and adolescents [62].

On the other hand, legal muscle-building supplements such as whey protein and creatine monohydrate are commonly used among young people. The most common protein sources used in sports supplementation are whey, casein from milk, ovalbumin from egg whites, legumes (soy and peas), and cereal proteins. Consuming protein-rich supplements particularly when timed around exercise, either pre- or post-workout, has been shown to significantly enhance muscle protein synthesis, supporting muscle growth and recovery [63].

Creatine is an endogenously produced compound, naturally synthesized in the body, and can also be obtained exogenously through dietary intake. It is essential for providing energy to our muscles and represents an important ergogenic aid among athletes. The use of creatine supplementation has significantly increased among adolescents over the past two decades, primarily to enhance athletic performance [64].

Several studies have shown that creatine supplementation has been linked to body weight gain. Creatine is an osmotically active substance, and this weight gain could be related to fluid retention and decreased diuresis during short-term use. Thus, this osmotically active effect could influence BP levels, warranting further investigation to elucidate its potential effect on cardiovascular health [65, 66].

## Conclusions

Research suggests that numerous unhealthy behaviors associated with cardiovascular disease often begin in childhood or adolescence [67, 68]. During these developmental stages, individuals may be particularly susceptible to the negative consequences of these behaviors. Moreover, the cumulative impact of unhealthy habits over time can significantly increase the risk of cardiovascular disease. Therefore, early intervention is crucial to prevent or mitigate these risks and promote lifelong cardiovascular health.

This review discusses several emerging cardiovascular risk factors that are becoming increasingly relevant in child health, including psychosocial stress, nicotine use, sleep disorders, excessive screen time, and energy drink consumption. These factors are interconnected by their negative impact on blood pressure. To our knowledge, no previous review has specifically addressed this relationship.

One significant limitation in evaluating individual habits is the predominant use of questionnaires as assessment tools. While many of the studies referenced in this review employ validated questionnaires, they are often subject to substantial information bias, particularly in pediatric populations, where parental reporting is likely. Implementing objective measures for these habits would significantly enhance the accuracy of assessments. For instance, accelerometry is widely used to assess physical activity, sleep duration, and sleep quality. Although the duration of mobile device usage can be measured, evaluating the specific content viewed remains challenging. Cotinine concentration serves as a reliable biomarker for nicotine consumption. Further advancements would involve correlating these objective measures with specific biomarkers indicative of endothelial injury or inflammation. This correlation would facilitate a comprehensive understanding of how these habits impact the cardiovascular health of the individuals studied.

Until we have biomarkers that help us stratify cardiovascular risk in the child population, interventions should focus on promoting healthy habits from an early age, stress management, limiting exposure to screens, promoting restful sleep, and education about the dangers of consuming substances such as nicotine and EDs. It is essential that prevention efforts focus on educating parents, educators, and young people themselves about the long-term effects of these habits and promote public policies that foster a healthy environment for the physical and mental development of the child and adolescent population.

In conclusion, pediatricians need to take a holistic view of children’s cardiovascular health, addressing not only traditional cardiovascular risk factors but also these non-traditional factors, emerging as significant threats. Early identification of these risks allows for implementing effective prevention strategies, encouraging healthy habits, and improving patients’ quality of life in the long term. Furthermore, by educating parents and children about the dangers of these habits, steps can be taken to reduce the risks of developing cardiovascular disease in the future.

## Data Availability

No datasets were generated or analysed during the current study.

## Abbreviations

ACEs: Adverse childhood experiences
AHA: American Heart Association
BMI: Body mass index
BP: Blood pressure
DBP: Diastolic blood pressure
EDs: Energy drinks
HTN: Hypertension
NYTS: National Youth Tobacco Survey
SBP: Systolic blood pressure
